# Oxidative stress inhibits axonal transport: implications for neurodegenerative diseases

**DOI:** 10.1186/1750-1326-7-29

**Published:** 2012-06-18

**Authors:** Cheng Fang, Dennis Bourdette, Gary Banker

**Affiliations:** 1 Jungers Center for Neurosciences Research, Department of Neurology, Oregon Health & Science University, Portland, Oregon, USA; 2 Multiple Sclerosis Center of Excellence-West Portland Veterans Affairs Medical Center, Portland, Oregon, USA

**Keywords:** Hydrogen peroxide, Oxidative stress, Axonal transport, Mitochondria, Golgi-derived vesicles, Neurodegeneration

## Abstract

**Background:**

Reactive oxygen species (ROS) released by microglia and other inflammatory cells can cause axonal degeneration. A reduction in axonal transport has also been implicated as a cause of axonal dystrophies and neurodegeneration, but there is a paucity of experimental data concerning the effects of ROS on axonal transport. We used live cell imaging to examine the effects of hydrogen peroxide on the axonal transport of mitochondria and Golgi-derived vesicles in cultured rat hippocampal neurons.

**Results:**

Hydrogen peroxide rapidly inhibited axonal transport, hours before any detectable changes in mitochondrial morphology or signs of axonal degeneration. Mitochondrial transport was affected earlier and was more severely inhibited than the transport of Golgi-derived vesicles. Anterograde vesicle transport was more susceptible to peroxide inhibition than retrograde transport. Axonal transport partially recovered following removal of hydrogen peroxide and local application of hydrogen peroxide inhibited transport, suggesting that the effects were not simply a result of nerve cell death. Sodium azide, an ATP synthesis blocker, had similar effects on axonal transport, suggesting that ATP depletion may contribute to the transport inhibition due to hydrogen peroxide.

**Conclusions:**

These results indicate that inhibition of axonal transport is an early consequence of exposure to ROS and may contribute to subsequent axonal degeneration.

## Background

Axonal transport is critical for maintaining axonal integrity. Anterograde axonal transport supplies the axon with proteins synthesized in the cell body and retrograde transport delivers endosomal signaling organelles from the terminal to the cell body [1]. Interrupting axonal transport leads to degeneration of the distal axon, much like the Wallerian degeneration that occurs after axonal transection [2]. Axonal transport is mediated by kinesins and dyneins, motor proteins that use ATP hydrolysis to power their translocation along microtubules [3,4]. Mutations that disrupt kinesin- or dynein-mediated transport lead to human diseases characterized by axonal dysfunction [5], such as some forms of Charcot-Marie-Tooth disease [6] and hereditary spastic paraplegia [7]. Alterations in axonal transport have also been implicated in neurodegenerative diseases, including Alzheimer’s disease [8], amyotrophic lateral sclerosis [9], Parkinson’s disease [10], and Huntington’s disease [11].

Oxidative stress, one of the major characteristics of neuro-inflammation, which results from the unregulated production of reactive oxygen species (ROS), has also been implicated in many neurodegenerative diseases [12-15]. Activated microglia, the resident innate immune cells in the brain, release ROS and reactive nitrogen species (RNS), including hydrogen peroxide, nitric oxide, peroxynitrite and superoxide [16]. Microglial activation occurs in many neurodegenerative diseases and in the neuroinflammatory diseases, such as multiple sclerosis [17,18]. Emerging evidence suggests that increased levels of ROS play an important role in triggering axonal degeneration [12-14]. Given the importance of axonal transport for the preservation of axonal integrity, surprisingly little is known about how oxidative stress affects axonal transport and whether this contributes to the damaging effects of elevated levels of ROS.

New methods based on live cell imaging make it possible to evaluate the efficiency of axonal transport with an increased level of precision [19]. We used this approach to investigate the effects of one ROS, hydrogen peroxide, a byproduct of mitochondrial oxidative metabolism that is elevated in many neurodegenerative diseases [20].

We found that hydrogen peroxide rapidly inhibited axonal transport of both mitochondria and Golgi-derived vesicles, providing the first direct evidence for the ability of ROS to inhibit axonal transport.

## Results

### Transport characteristics of mitochondria and Golgi-derived vesicles

**Figure 1:**
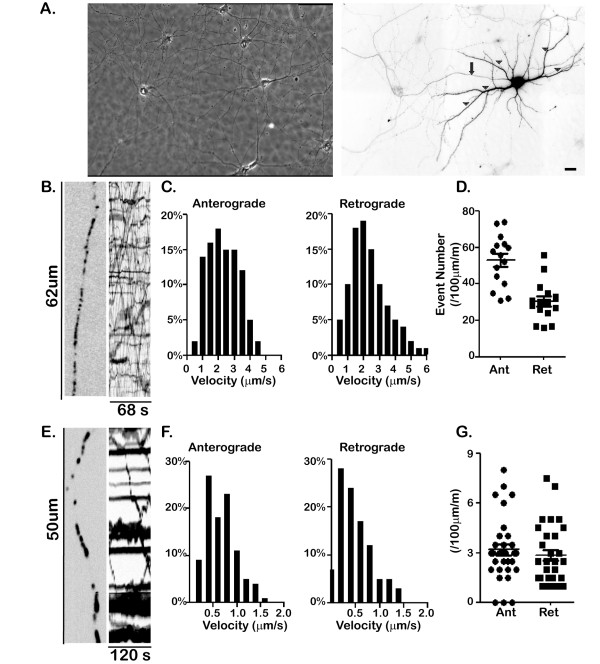
**Characteristic features of the transport of mitochondria and Golgi-derived vesicles.A**. A phase contrast image shows the extensive network formed by neurons after 7 days in cultures (left panel). The morphology of a single transfected neuron is shown by soluble GFP (right panel, color inverted), making it possible to follow the course of the axon (arrow) and dendrites (arrow-heads). We imaged axonal transport in the proximal part of the axon, beginning 30–50 μm beyond the initial segment. Scale bar, 20 μm. **B-D**. Axonal transport of Golgi-derived vesicles. **B**. The left panel shows an enlargement of a stretch of axon from a cell transfected with a marker for Golgi-derived vesicles (contrast inverted) and the right panel shows a kymograph quantifying the transport of Golgi-derived vesicles transport in this axon. Golgi-derived vesicles are small and round shaped vesicles. Vesicles undergoing anterograde transport correspond to diagonal lines with positive slope in the kymograph (see Methods for additional details). Horizontal lines represent stationary vesicles. **C**. Histograms showing the transport velocities of Golgi-derived vesicles (based on 772 events in 15 cells). Anterograde transport (2.1 ± 1 μm/sec) is significantly faster than retrograde transport (1.6 ± 0.81 μm/sec) (Student t-test, P < 0.001). Error bar indicates SD. **D**. A scatter plot showing the anterograde and retrograde event numbers (number of events/100 μm/minute) for each axon. Golgi-derived vesicles anterograde transport event numbers range from 24 to 78 during the one minute imaging time while the retrograde event numbers only range from 16 to 60 events per minute (anterograde, circle; retrograde, square). The average anterograde to retrograde event ratio was 1.73. **E-G**. Axonal transport of mitochondria. **E**. The left panel is an enlargement of a stretch of axon from a cell expressing mitoGFP (contrast inverted) and the right panel is the kymograph illustrating mitochondrial transport in this axon. **F**. Histograms showing the velocity of mitochondrial transport (based on 321 events from 32 cells). The velocity of mitochondrial transport is similar in both directions (anterograde 0.6 ± 0.41 μm/sec vs. retrograde 0.59 ± 0.35 μm/sec, student t-test, P > 0.9). **G**. A scatter plot showing the number of anterograde and retrograde movements of mitochondria. Mitochondrial transport had no directional bias, with both anterograde and retrograde event numbers ranging from 0 to 8 events/100 μm/minute. The average anterograde to retrograde event ratio was 1.01 for mitochondria. Error bar indicates SD.

Mitochondria and Golgi-derived vesicles are two principal classes of organelles moved by axonal transport. We analyzed the axonal transport of mitochondria and Golgi-derived vesicles in hippocampal neurons, following expression of suitable GFP-tagged constructs (Figure 1). Golgi-derived vesicles moved at rapid rates (2.1 ± 1 μm/sec anterograde and 1.6 ± 0.8 μm/sec retrograde), often traversing long distances without pause. Many vesicles moved in both directions, but the majority of movements were in the anterograde direction (ratio of anterograde to retrograde events: 1.73:1). The transport of mitochondria was distinctly different than vesicles. Only a minority of the labeled mitochondria moved during the 2-minute period of imaging and the rate of their movement was distinctly slower than that of Golgi-derived vesicles (0.60 ± 0.41 μm/sec anterograde and 0.59 ± 0.35 μm/sec retrograde). An equal number of mitochondria moved in both directions; individual movements were shorter and there were more frequent pauses.

### Hydrogen peroxide-induced inhibition of axonal transport occurs hours before the first signs of axonal degeneration

**Figure 2:**
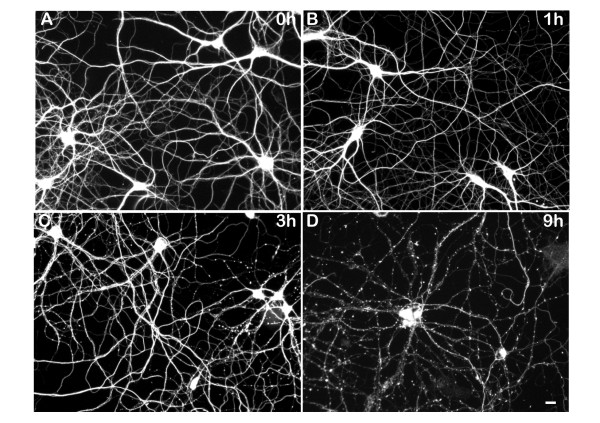
**The time course of axonal degeneration for hydrogen peroxide exposure.** Neurons were treated with 100 μM hydrogen peroxide (or PBS as control) for up to 24 h, and axonal morphology was monitored by neuron-specific beta3-tubulin staining. After one hour, cells remained normal (**B**). After 3 h, many axons started to show signs of degeneration as indicated by swelling of the axons (**C**); by 9 h, the axons exposed to hydrogen peroxide were completely fragmented (**D**). In contrast, neurons in the mock-treated culture remained intact even 24 h later. Scale bar, 20 μm.

**Figure 3:**
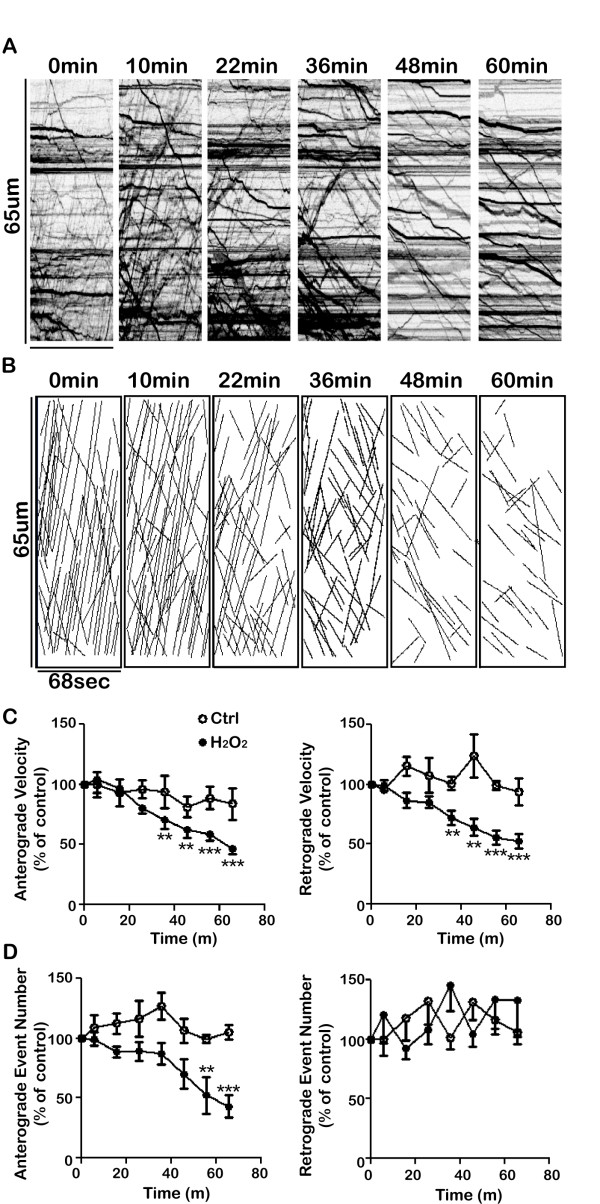
**Hydrogen peroxide inhibits vesicle transport.A**. Representative kymographs illustrating the transport of Golgi-derived vesicles before and at various time points after exposure to 100 μM hydrogen peroxide. **B**. Hand-traced lines from the above kymographs. **C&D**. Quantitative data of Golgi-derived vesicle transport following hydrogen peroxide treatment (n = 20 cells). For each cell, the velocity and event number were normalized to pretreatment levels. **C**. Velocities of Golgi-derived vesicles in both directions decreased over time. **D**. The anterograde event numbers of Golgi-derived vesicles was inhibited to around 40% by the end of one hour while the number of retrograde events did not change. Under this imaging condition, both velocities and event numbers of Golgi-derived vesicles transport in control neurons remain stable (control, open circle; hydrogen peroxide treatment, filled circle). The transport at each time point was compared with pre-treatment levels using paired student t-test (* P < 0.05; ** P < 0.01; *** P < 0.001). Error bar indicates SD.

Prolonged exposure to hydrogen peroxide leads to axonal degeneration [21]. We set out to determine whether inhibition of axonal transport was an early or a late event in this process and whether there were differences in how hydrogen peroxide affected the transport of different organelles. We first examined the time course of axonal degeneration. As shown in Figure 2, axon beading, an early sign of damage, occurred after continuous exposure to hydrogen peroxide for 3 hours. After 9 hours, most axons were completely fragmented. Based on these results, we examined the effects of hydrogen peroxide on axonal transport during the first hour of exposure, well before the first evidence of microtubule damage based on tubulin staining. Figure 3A shows a series of kymographs that illustrate changes of vesicle transport in one axon due to hydrogen peroxide exposure. Prior to treatment, there was abundant transport in this axon (34 anterograde events and 23 retrograde events over the one minute imaging time). After 22 minutes, there was a reduction in the velocity of both anterograde and retrograde transport (anterograde: from 2.1 μm/s to 1.0 μm/s; retrograde: from 1.2 μm/s to 0.8 μm/s). There was also a marked reduction in the number of anterograde movements but the number of retrograde events remained unchanged. By 60 minutes, there were only 5 anterograde events left in that one minute imaging period. Thus the anterograde to retrograde event ratio for this neuron declined from 1.48 to 0.18 during hydrogen peroxide exposure.

Figure 3C and 3D summarize the effects of hydrogen peroxide on vesicle transport, based on an analysis of 20 cells. Hydrogen peroxide caused a significant reduction in the velocity of both anterograde and retrograde transport (which reached statistically significant levels after about 35 minutes of exposure) and in the number of anterograde transport events (which reached statistical significance after 50 minutes). There was no reduction in the number of retrograde events. This “signature” of hydrogen peroxide damage—a reduction in the velocity of both anterograde and retrograde transport but a selective reduction in the number of anterograde events—was apparent in the recordings from nearly every cell. Since anterograde transport was preferentially inhibited, the anterograde over retrograde ratio fell from a mean of 1.7:1 before exposure to 0.7:1 after exposure for 1 h.

**Figure 4:**
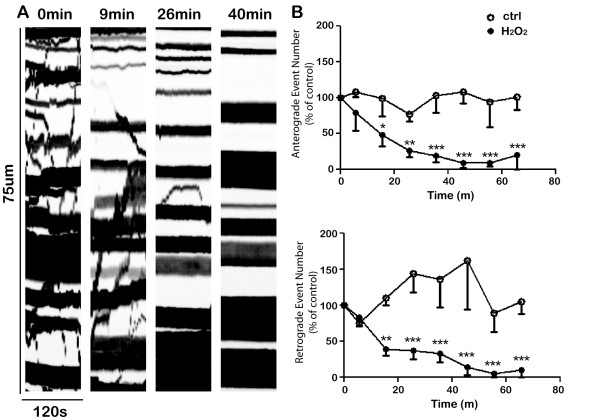
**Hydrogen peroxide inhibits mitochondrial axonal transport.A**. Representative kymographs of mitochondrial transport at varying times after exposure to 100 μM hydrogen peroxide. **B**. Quantitative analysis of mitochondrial transport following hydrogen peroxide treatment (n = 20 cells). The number of anterograde and retrograde events (normalized to each cell’s pretreatment levels) decreased over time (control, open circle; hydrogen peroxide treatment, filled circle). The transport at each time point was compared with pre-treatment levels using paired student t-test (* P < 0.05; ** P < 0.01; *** P < 0.001). Error bar indicates SD.

We next examined the effects of hydrogen peroxide on mitochondrial transport (Figure 4). Hydrogen peroxide led to a profound inhibition of mitochondrial transport. This effect was already obvious after only 9 minutes of exposure and by 26 minutes only minimal transport could be observed. As summarized in Figure 4B (based on analyses of 20 cells), the number of anterograde and retrograde events was reduced by 50% within about 16 minutes of exposure to peroxide and by 1 h the amount of transport was reduced to less than 10% of control. There also appeared to be some reduction in the velocity of mitochondrial transport after about 30 minutes, but by this time there were so few events that it was difficult to accurately obtain an average velocity.

A comparison of Figure 3D and 4B clearly indicates that hydrogen peroxide has very different effects on the transport of Golgi-derived vesicles compared with mitochondria. Mitochondrial transport was much more sensitive to the effects of hydrogen peroxide and anterograde and retrograde mitochondrial transport were both affected similarly. Inhibition of vesicle transport required longer exposures to hydrogen peroxide and anterograde vesicle transport was more severely affected.

As expected, the effects of hydrogen peroxide on axonal transport were dose-dependent. At higher concentrations (250 μM hydrogen peroxide), the inhibition of transport occurred more rapidly and the number of vesicles moving in both the anterograde and retrograde directions was significantly reduced. As at lower concentrations of hydrogen peroxide, anterograde vesicle transport was more severely affected. Anterograde transport was inhibited by 70% after about 35 minutes, while a comparable inhibition of retrograde transport required exposure for 70 minutes. Lower concentrations of hydrogen peroxide had less dramatic effects. Exposure to 20 μM and 50 μM hydrogen peroxide inhibited anterograde vesicle transport by 20% and more than 30% respectively after 1 hour and had no effects on retrograde transport in both concentrations. For any given concentration of hydrogen peroxide, mitochondrial transport was more sensitive to hydrogen peroxide than was the transport of Golgi-derived vesicles.

### Local exposure to hydrogen peroxide inhibits axonal transport

**Figure 5:**
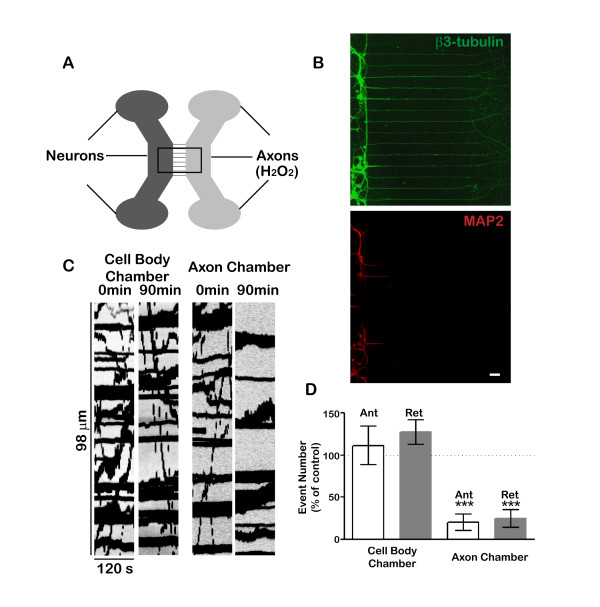
**Local hydrogen peroxide exposure inhibits axonal transport.** Neurons were cultured in Xona® microfluidic chambers, which permit independent control of the fluid environment surrounding the axonal and somato-dendritic compartments. (**A**). Neurons were seeded in the cell body chamber. After 5–10 days, their axons extended through the microchannels and into the axon chamber. The length of microchannels (450 μm) ensures that only axons extend far enough to enter the other side. Beta3-tubulin staining (green) and MAP2 staining (red) confirmed that cell bodies and dendrites were confined in the cell body chamber and only axons extended into the axon chamber (**B**). Mitochondrial transport was imaged in both the cell body and axon chambers before treatment. Then hydrogen peroxide (100 μM) was added into the axon chamber. Mitochondrial transport in axon chamber was severely inhibited 90 m after hydrogen peroxide treatment while transport in cell body chamber was largely intact (**C**). **D**. Quantification showed that transport in the axonal compartment was inhibited by more than 80% (*** P < 0.001 paired student t-test). Error bar indicates standard error.

The effects of hydrogen peroxide on transport could be due to direct, local effects on the axonal transport machinery or to indirect effects that follow damage to neuronal cell bodies and dendrites. To explore these two possibilities, we grew hippocampal neurons in microfluidic chambers [22] that enable independent manipulation of the fluid environment surrounding cell bodies and axons, as illustrated in Figure 5. Before addition of hydrogen peroxide, there was extensive mitochondrial transport in both the cell body and axonal compartments. One hour after adding hydrogen peroxide to the axonal chamber, mitochondrial transport was severely inhibited, while transport in the cell body compartment was unaffected (Figure 5C&D). This strongly suggests that hydrogen peroxide’s effect on axonal transport is a direct effect on the axon rather than an indirect consequence of damage to the cell body. Because only about 1% of cells express SS-mCherry following transfection and only a small fraction of axons pass through the microchannels, it was not possible to investigate local effects of hydrogen peroxide on the transport of Golgi-derived vesicles.

### Axonal transport recovers partially after hydrogen peroxide exposure

**Figure 6:**
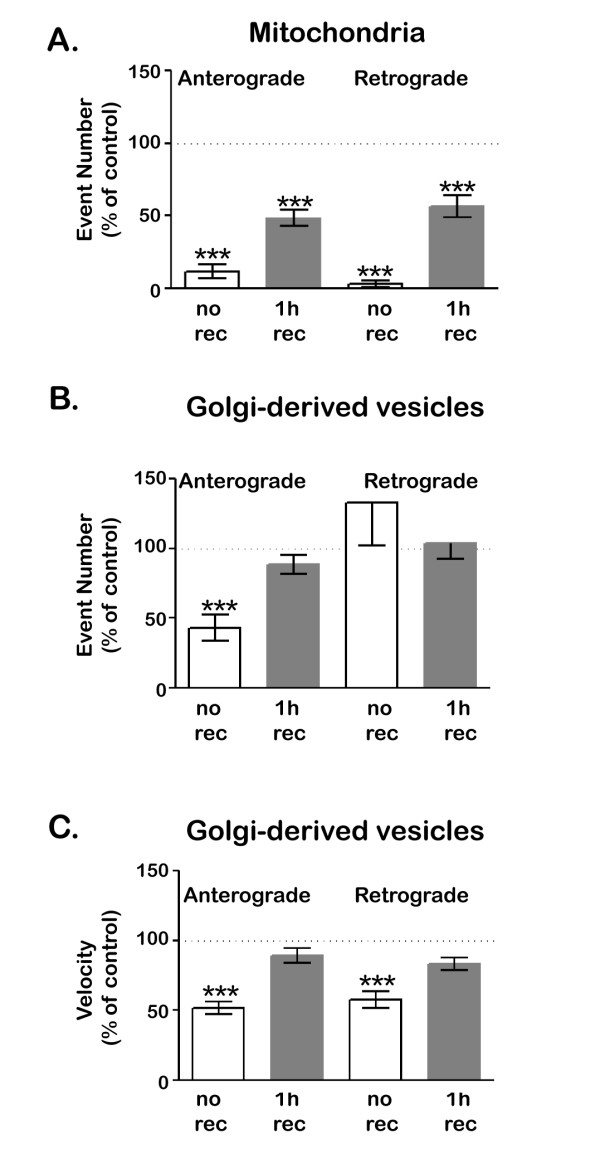
**Axonal transport partially recovers after exposure to hydrogen peroxide.** Transport was imaged in cultures that were exposed to 100 μM hydrogen peroxide for 1 h, that were exposed for 1 h then allowed to recover for 1 h, or that were mock-treated. **A**. After recovery, mitochondrial transport remained significantly reduced compared to control cells (*** P < 0.001, student t-test). In the case of Golgi-derived vesicles, both the number of transport events (**B**) and the velocity of transport (**C**) fully recovered after hydrogen peroxide treatment (P > 0.5, student t-test). Error bar indicates SD.

We next sought to determine whether the inhibition of axonal transport caused by exposure to hydrogen peroxide was permanent (Figure 6). As seen in previous experiments, exposure to hydrogen peroxide for 1 h reduced the number of mitochondrial movements in both anterograde and retrograde directions to less than 10% of control levels (Figure 6A) After cultures were returned to control medium for one hour, mitochondrial transport remained inhibited by about 50%. Exposure to lower concentrations of hydrogen peroxide (50 μM) reduced mitochondrial transport less (to about 40% of control levels) and allowed greater recovery (to over 80% of control levels; data not shown).

In contrast to mitochondrial transport, anterograde transport of Golgi-derived vesicles, which dropped by about 50% after treatment with 100 μM hydrogen peroxide, was fully restored after recovery for 1 hour (Figure 6B). Moreover, the velocities of anterograde and retrograde vesicle transport also returned to control levels after 1 hour recovery (Figure 6C). The recovery of vesicle transport was also dose-dependent. Following exposure to 250 μM hydrogen peroxide, anterograde transport of Golgi-derived transport was reduced to less than 20% of controls and retrograde transport was also reduced (to about 30% of controls). Transport of Golgi-derived vesicles recovered partially after return to control media for 1 hour, but failed to reach control levels. Retrograde transport of Golgi-derived vesicles was less severely inhibited than anterograde transport and recovered more completely (data not shown). Taken together, these data suggest that axonal transport recovers from mild but not from severe hydrogen peroxide–induced inhibition. For any given concentration of hydrogen peroxide, mitochondrial transport is more sensitive to hydrogen peroxide than is the transport of Golgi-derived vesicles and is less likely to recover.

### Hydrogen peroxide-induced axonal degeneration precedes neuronal cell death

**Figure 7:**
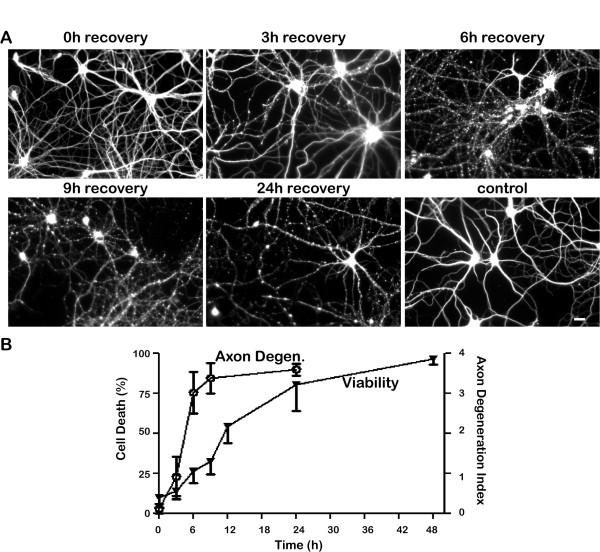
**Hydrogen peroxide exposure leads to irreversible axonal degeneration.A**. Neurons were treated with 100 μM hydrogen peroxide for one hour, and then returned to control media immediately after exposure. Cell morphology remained normal right after the exposure. However, signs of axonal degeneration were noted within a few hours after treatment and most axons were completely degenerated by 24 h. Scale bar, 20 μm. **B**. The degree of axonal degeneration was scored basing on axonal morphology (Y-axis, right side) and cell death was assayed by staining of ethidium homodimer and rejection of calcein AM (Y-axis, left side). Axonal degeneration clearly preceded neuronal death. For control neurons, the degeneration score was less than 1 and the percentage of cell death was less than 10% even at 24 h after mock treatment. Degeneration scores are based on analysis of more than 90 regions and cell death analyses from counts of more than 300 cells in three independent experiments. Error bar indicates SD.

Since axonal transport did not fully recover after exposure to higher concentrations of hydrogen peroxide, even though axonal morphology appeared to be normal right after exposure, we wondered about the long-term consequences of this treatment for axonal integrity. To examine this question, we exposed cultured hippocampal neurons to100 μM hydrogen peroxide for one hour, then returned them to control medium for varying periods of time. Axonal integrity was evaluated by immunostaining neuronal microtubules. Figure 7A illustrates the changes in axonal morphology that were observed at varying times after only one hour exposure of hydrogen peroxide and Figure 7B quantifies the effects on axonal integrity and cell survival. At the end of the 1 hour exposure to hydrogen peroxide, axons appeared entirely intact, but by 3 hours post-treatment early signs of damage, such as beading, could be detected. Extensive axonal fragmentation occurred 6–9 hours after treatment. Neuronal cell death was significantly delayed compared with axonal degeneration, occurring 12–48 hours after exposure to hydrogen peroxide. These observations are consistent with several other findings indicating that axonal degeneration occurs long before cell death following oxidative stress [21,23,24].

Changes in mitochondrial morphology have been reported to correlate with axonal damage and are thought to be one of the earliest signs of axonal degeneration, preceding changes in axonal morphology [25]. We wondered whether hydrogen peroxide caused changes in mitochondrial morphology prior to its effects on axonal transport. Mitochondrial morphology was evaluated by computing the shape factor, an indicator of how similar an object is to a perfect sphere, which has a shape factor of 1.0. Even after 1 hour of exposure to hydrogen peroxide, when mitochondrial transport was reduced by 90%, there was no change in mitochondrial shape (shape factor: control 0.78 ± 0.19; 100 μM hydrogen peroxide for 1 hour 0.78 ± 0.22; n = 173 and 147 mitochondria, respectively). These data show that axonal transport inhibition precedes changes in mitochondrial morphology.

**Figure 8:**
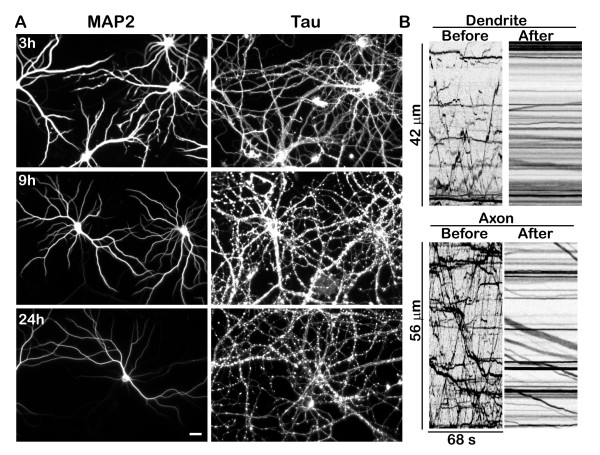
**Axons are more sensitive to hydrogen peroxide than dendrites.A**. Neurons were treated with 100 μM hydrogen peroxide for one hour, and then returned to control medium. At varying time points, they were fixed and stained with antibodies against MAP2 (a dendritic marker) and Tau (an axonal marker). Axons started degenerating at 3 h after treatment and mostly degenerated by 9 h (right panel). In contrast, dendrites of surviving cells remained intact even 24 h after treatment (left panel). Scale bar, 20 μm. **B**. Kymographs illustrating the transport of Golgi-derived vesicles in the axon and dendrite of the same neuron before and after one hour of hydrogen peroxide treatment. The transport of Golgi-derived vesicles was severely inhibited in dendrites as well as axons.

To further investigate differences in the effects of hydrogen peroxide exposure on axons versus cell bodies and dendrites, we exposed cultures to hydrogen peroxide for 1 hour, and then examined the morphology of their axons and dendrites by staining with antibodies specific for axons (anti-tau) and cell bodies and dendrites (anti-MAP2). The results of this experiment are illustrated in Figure 8. Axonal beading was evident in anti-tau stained cultures as early as 3 hours after hydrogen peroxide exposure and most axons were fragmented by 9 hours. Despite the extensive axonal degeneration, the dendritic arbors of surviving cells looked remarkably normal. Even after 24 hours, when the axonal plexus appeared completely degenerated and most cells had died, the dendritic arbors of the surviving cells appeared intact. It is clear from these results that dendrites are far more resistant to hydrogen peroxide damage than are axons.

Given these results, we wondered whether dendritic transport of mitochondria and Golgi-derived vesicles was less sensitive to hydrogen peroxide than axonal transport. As shown in Figure 8B, the transport of Golgi-derived vesicles was profoundly inhibited in both the dendrites and the axon. These results suggest that the initial effects of hydrogen peroxide exposure are similar in axons and dendrites, but the long-term consequences of this exposure preferentially affect the axon, ultimately leading to its degeneration.

### ATP depletion reversibly inhibits axonal transport and does not induce axon degeneration

Axonal transport is ATP-dependent [26] and *in vitro* assays show that the motors that mediate axonal transport hydrolyze 1 molecule of ATP for every step they take along the microtubule [27]. Since hydrogen peroxide damages mitochondria and causes depletion of ATP [28], we set out to determine whether ATP depletion contributes to the inhibition of axonal transport and the axonal degeneration that occur following hydrogen peroxide exposure. To address this issue, we treated hippocampal cultured neurons with sodium azide (NaN_3_), a specific inhibitor of cytochrome C oxidase, complex IV of the mitochondrial respiratory chain.

**Figure 9:**
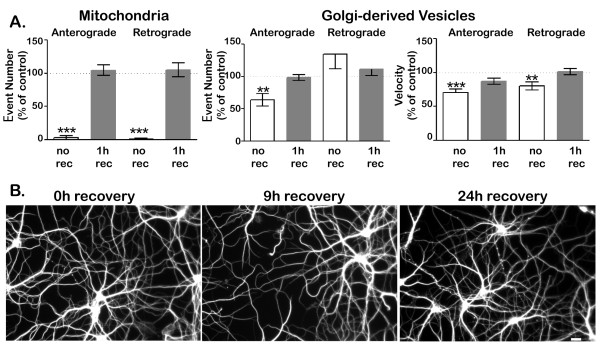
**Sodium azide treatment reversibly inhibits axonal transport but does not cause axonal degeneration.A**. Neurons were treated with 1 mM sodium azide for 1 h, with sodium azide for 1 h and then returned to control media for 1 h, or were mock-treated. Axonal transport of both Golgi-derived vesicles and mitochondria was severely inhibited by sodium azide, but recovered completely. Values for transport events and velocity were normalized to control level. Error bar indicates SD. **B**. Axonal morphology was visualized at varying times after 1 h sodium azide exposure using antibodies against beta3-tubulin. Exposure to 1 mM sodium azide for 1 h did not induce axonal degeneration. Scale bar, 20 μm. (* P < 0.05; ** P < 0.01; *** P < 0.001).

Cultured hippocampal neurons were exposed to 1 mM sodium azide, a concentration that does not cause mitochondrial ROS production, as tested by amplex assays (Invitrogen) (data not shown). The transport of both mitochondria and Golgi-derived vesicles was inhibited by sodium azide (Figure 9A). At the end of one hour, both anterograde and retrograde mitochondrial transport was reduced to less than 10% of control values. The anterograde transport of Golgi-derived vesicles was reduced to 60% of control, while the retrograde transport of Golgi-derived vesicles was not inhibited. These data show that the effects of ATP depletion on axonal transport are similar to those of hydrogen peroxide. Mitochondrial transport was more profoundly affected than vesicle transport and vesicle transport was preferentially inhibited in the anterograde direction.

We also examined whether the axonal transport returned to normal levels following ATP depletion. Following exposure to azide for 1 h, the transport of both mitochondria and Golgi-derived vesicles (velocity as well as event numbers) recovered completely (Figure 9A). This is quite different from the effects of hydrogen peroxide treatment, which permits only a partial recovery of mitochondrial axonal transport. Moreover, one hour of sodium azide treatment did not induce axonal degeneration. Axons remained intact even at 24 hours after treatment, as shown by beta3-tubulin staining (Figure 9B). Taken together, these data suggest that ATP depletion may contribute to some of the effects of hydrogen peroxide on axonal transport, but the latter agent leads to irreversible changes in the machinery underlying axonal transport that are not due simply to ATP depletion.

## Discussion

This report is the first direct demonstration of the ability of hydrogen peroxide to inhibit axonal transport. Using live-cell imaging of cultured hippocampal neurons, we found that hydrogen peroxide profoundly inhibited the axonal transport of both mitochondria and Golgi-derived vesicles. Surprisingly, mitochondrial transport was affected earlier and was more severely inhibited than vesicle transport.

The paradigm we used—live-cell imaging of cultured neurons--allowed us to rigorously characterize the effects of hydrogen peroxide on axonal transport, but it required that we use comparatively high concentrations, so that the effects on transport could be observed within an hour or so. These concentrations of hydrogen peroxide eventually lead to axonal degeneration and ultimately nerve cell death, but several lines of evidence suggest that the hydrogen peroxide-induced inhibition of axonal transport we observed is not simply the response of a dying cell. First, the inhibition of transport occurred relatively early, even before a change in mitochondrial morphology, which is thought to be among the earliest signs of ROS-induced axonal damage [25]. Second, the effects were seen after local exposure of axons to hydrogen peroxide in microfluidic chambers in which the nerve cell bodies are not exposed to hydrogen peroxide. Third, after removal of hydrogen peroxide, vesicle transport returned to near-normal levels. If the inhibition of transport was a secondary consequence of axonal degeneration, one would expect it to worsen over time, not to improve. Fourth, different components of axonal transport were differentially affected. It seems unlikely that transport failure secondary to axonal degeneration would inhibit anterograde vesicular transport but spare retrograde transport. Finally, similar but milder effects on axonal transport were observed during a 1-hour exposure to lower concentrations of peroxide. Under these conditions, the great majority of axons show no evidence of morphological damage one day after treatment.

### How does hydrogen peroxide exposure lead to inhibition of transport?

Hydrogen peroxide, like other ROS, disrupts many cellular processes, including mitochondrial ATP production and regulation of calcium homeostasis [29], ion channel permeability [30], and redox signaling [31]. At present, we do not know the pathways that lead to inhibition of axonal transport. Some of the effects of hydrogen peroxide on axonal transport were quite similar to those produced by sodium azide, an inhibitor of ATP production [27]. Following both treatments, mitochondrial transport was inhibited first, then anterograde vesicle transport, and then retrograde vesicle transport. Thus it is reasonable to attribute some of the effects of hydrogen peroxide exposure to ATP depletion. Several of our findings, however, suggest that there is more involved than simply a reduction in the ATP levels available to molecular motors. Since kinesins and dyneins both have similar requirements for ATP, the differential effects on anterograde versus retrograde transport are more likely to involve the many signaling pathways activated by increased levels of ROS than simply a reduction in ATP. For example, stress-activated kinases [32] can phosphorylate kinesins and inhibit their translocation, which could lead to a selective inhibition of anterograde transport.

Why is mitochondrial transport preferentially inhibited by hydrogen peroxide and sodium azide? One possibility relates to the molecular adaptors that link mitochondria to kinesins and enable cytoplasmic calcium levels to regulate mitochondrial transport. Miro, an EF-hand containing protein in the outer mitochondrial membrane, binds to Milton, a cytoplasmic adaptor protein that in turn binds to the heavy chain of Kinesin-1 [33,34]. When cytoplasmic calcium levels increase, calcium binds to the EF hands on Miro, permitting Miro to bind to the motor domain of kinesin-1 and stopping mitochondrial transport [35,36]. Both hydrogen peroxide treatment and ATP depletion disrupt mitochondrial calcium buffering and elevate cytoplasmic calcium levels [29,30], which would be expected to activate Miro. In contrast, Golgi-derived vesicles are linked to Kinesin-1 via interactions that are not calcium-dependent.

We focused our work on the effects of hydrogen peroxide because it is elevated in some models of neuroinflammatory and neurodegenerative diseases [37-39] and because it is easy to manipulate *in vitro*. However, based on similarities in their mechanisms of action, it would not be surprising if other ROS and RNS had similar effects on axonal transport. Stagi et al [32] reported that exposure to nitric oxide inhibited axonal transport of synaptic vesicle proteins in cultured hippocampal neurons, although the fluorescence correlation spectroscopy method they used did not allow characterization of the effects at the level of individual vesicle movements. Although the molecular mechanism of hydrogen peroxide-induced axonal transport inhibition is not clear, activation of stress-activated kinases can phosphroylate kinesins, inhibiting axonal transport [40,41]. Activation of local apoptotic pathways could also be involved [42].

### Could ROS-induced inhibition of transport contribute to axonal disease?

Our results indicate that hydrogen peroxide exposure preferentially inhibits mitochondrial trafficking and the anterograde transport of Golgi-derived vesicles, including those that carry proteins essential for maintaining axonal integrity [2]. Retrograde transport, which delivers trophic factors required for cell survival, is less affected. Over the long term, if axons were exposed to local increases in ROS species, this pattern of transport inhibition could result in the dying-back axonal degeneration that characterizes several neurodegenerative diseases, including the progressive phase of some neuroinflammatory diseases. Our results also show that axons are more susceptible to ROS damage than nerve cell bodies and dendrites, so it is possible that more global changes in inflammation, which are hypothesized to play a role in several neurodegenerative diseases [12-15], could preferentially affect axons.

While there may be some situations in vivo where axons are exposed to high concentrations of hydrogen peroxide or other ROS [25], in most diseases that have an inflammatory component axons are likely to be chronically exposed to modestly increased levels of ROS. It remains to be seen if the effects we observed following acute exposure to hydrogen peroxide are mimicked during chronic exposure to lower levels of this agent or other ROS. To the extent it was possible to examine the dose–response characteristics in our model, we observed similar effects at lower concentrations of hydrogen peroxide, but they were less severe and occurred over a slower time course. If ROS-mediated inhibition of axonal transport contributes to neural disease in vivo, the cell culture model we describe here for analyzing axonal transport could serve to elucidate the molecular details of how ROS disrupt axonal transport and suggest potential neuroprotective therapies.

## Conclusions

In this study, we show that exposing cultured hippocampal neurons to hydrogen peroxide, one of the common ROS elevated during inflammation, causes an inhibition of axonal transport several hours before any signs of axonal degeneration occur. The transport of different organelles was differentially affected—mitochondrial transport was affected earlier and more severely than the transport of Golgi-derived vesicles; anterograde transport of Golgi-derived vesicles was inhibited more severely than retrograde transport. Local exposure of axons to hydrogen peroxide was sufficient to inhibit axonal transport and induce axonal degeneration. These results raise the possibility that ROS and RNS-induced inhibition of axonal transport in apparently healthy axons could contribute to the dying-back axonal degeneration that occurs in some neurodegenerative diseases.

## Materials and methods

### Primary neuronal culture and transfection

Primary hippocampal cultures were prepared from E18 embryonic rats of either sex as described previously [43,44] Cells were plated at 75 cells/mm^2^ on poly-L-lysine-treated coverslips and maintained in MEM supplemented with N2. For electroporation, 0.5-1 μg of DNA was introduced into the neurons through electroporation at DIV0 before seeding and then the neurons were seeded at 500 cells/mm^2^. Neurons were imaged at DIV7-10. For transfection, 1–2 μg of DNA was introduced to the neurons at 7–8 days in vitro (DIV) using Lipofectamine 2000 (Invitrogen, Carlsbad, CA). Neurons were imaged 16–20 hours after transfection.

### Immunostaining

Live-dead assay was performed following company protocol (Invitrogen, Carlsbad, CA). Briefly, Calcein AM and EthD-1 were added directly to neuronal culture medium (final concentration 2 μM of calcein AM and 4 μm of EthD-1). Cells were returned to the incubator for 20 minutes. Following incubation, cells were gently rinsed with warm PBS and changed into fresh neuronal culture medium and immediately brought to the microscope. Cells were maintained at 37 degrees for live imaging.

For immunostaining, DIV7-10 neurons were fixed in 4% paraformaldehyde, 4% sucrose for 15 minutes, permeabilized in 0.25% Triton X-100 for 5 minutes prior to primary antibodies (Tuj-1, Sigma; Map2b, Sigma; Tau-1, Sigma) 4 degrees overnight followed by secondary antibodies (488 anti Mouse, Cy3 anti Rabbit, Jackson ImmunoResearch Laboratories, West Grove, PA) at room temperature for 45 minutes. The coverslips were mounted in elvanol [43].

### DNA constructs

All constructs were expressed from the plasmid vector containing a beta-actin promoter/enhancer [45]. SS-mCherry was engineered to have secretory sequence of neuropeptide Y (NPY) [46] fused to the N terminus of red fluorescent protein mCherry. Mito-GFP was constructed by insertion of a mitochondria targeting sequence from subunit VIII of human cytochrome c oxidase to the N terminus of eGFP. All constructs were verified by sequencing.

### Imaging and analysis

Before acquiring movies, coverslips were semi-sealed into a heated chamber (Warner instruments, Hamden, CT) containing hibernate A medium (Brainbits LLC, Springfield, IL) kept at 37 degree. Images were captured with a spinning disk confocal microscope setup custom built by Solamere Technology Group (Salt Lake City, Utah). Laser excitation wave length for different fluorophores was 488 nm for GFP and 568 nm for mCherry. Mitochondrial transport was acquired using a 40X 1.3 N.A oil objective. Golgi-derived vesicles transport was acquired using a 60X 1.45 N.A oil objective. Both objective and the imaging stage were heated to 37 degrees.

To perform analysis of organelle transport, we used kymograph function in Metamorph. For each movie frame, the brightest pixel within a 2 μm corridor along the axis of an axon (or dendrite) is displayed at the corresponding location of a kymograph. The fluorescence patterns for all frames are then displayed adjacent to one another. This produced a graph on which the x-axis represents time and the y-axis represents distance along the process. The diagonal lines on each kymograph indicated moving organelles and were traced on each kymograph. All organelles that moved more than 5 μm were included in the data. Event numbers were computed by the total amount of transport numbers in the stretch of axon of interest and then were normalized to every 100 μm. Statistical significance of differences between groups was determined by performing student t-test using statistical software Prism®.

Mitochondrial morphology from the same stretch of axon was examined before hydrogen peroxide treatment and at 30 min and 60 min after treatment. Regions with no or few overlapping mitochondria were picked. Mitochondrial morphology was measured as shape factor (SF) using Metamorph. SF = 4π A/P2 (P = perimeter and A = area). SF gives a value from 0 to 1 representing how closely the object represents a circle: a value near 0 indicates a flattened object, whereas a value of 1 indicates a perfect circle.

### Axonal degeneration score

Axon morphology was judged by the beta3-tubulin staining of neurons. Intact axons, characterized by their thin and uniform diameter, were determined as score 0 in our axonal degeneration system. When some axonal swelling was obvious but the majority of the axons were still intact, we considered that score 1. When almost half of the axons were swelling but there was no or minimal axonal fragmentation, we considered that score 2. Score 3 indicated most of the axons in the field were swelling and some were fragmented. Finally, if all the axons were fragmented, that was considered score 4. Beta3-tubulin staining pictures of cells before and at various time points after treatment (or control) were judged by experimenter blind to the treatment conditions based on the scoring system described above. Each condition was repeated 3 times.

## Competing interests

The authors declare that they have no competing interests.

## Authors’ contributions

CF participated in experimental design, carried out all the experiments described and drafted the manuscript. GB and DB were involved in the design of experiments and production of the manuscript. All authors participated in revising and editing the final manuscript. The final manuscript was read and approved by all authors.
